# A finite element analysis of relationship between fracture, implant and tibial tunnel

**DOI:** 10.1038/s41598-021-81401-6

**Published:** 2021-01-19

**Authors:** Yiqun Wang, Erpeng Qi, Xiaojun Zhang, Lu Xue, Lianyou Wang, Jiahe Tian

**Affiliations:** 1 Medical School of Chinese PLA, Chinese PLA General Hospital, Beijing, China; 2 Department of Nuclear Medicine, Chinese PLA General Hospital, 28 Fuxing Road, Beijing, China; 3 Department of Orthopaedics, Tianjian Hospital, Tianjin, China; 4 Department of Pathology, China-Japan Union Hospital of Jilin University, No. 126 Xiantai Street, Changchun, Jilin China

**Keywords:** Trauma, Fracture repair

## Abstract

The purpose of this article was to use finite element analysis (FEA) to study the relationship of tibial tunnel (TT) with fracture pattern and implants. A computed tomography scan of full-length tibia and fibula was obtained. Models were built after three-dimensional reconstruction. The corresponding plates and screws were constructed and assembled together with fracture models. FEA was performed and contourplots were output. The Von Mises stresses of nodes and displacements of elements were extracted. Student’s t test was used to compare the values of Von Mises stresses and displacements between corresponding models. Differences in Von Mises stresses and displacements of fragments and implants between models with and without TT were nearly all statistically significant. However, the displacements of fragments and implants for all models were < 2 mm. TT in fracture models had larger Von Mises stresses than TT in intact tibial model. However, displacements of TT in fracture models showed similar or even smaller results to those in intact tibial model. Although almost all the tested parameters were statistically significant, differences were small and values were all below the clinical threshold. This study could promote open reduction and internal fixation with one-stage reconstruction for treatment of tibial plateau fractures associated with anterior cruciate ligament (ACL) ruptures.

## Introduction

Tibial plateau fractures (TPF) account for about one-tenth of tibial fractures^1^, and isolated anterior cruciate ligament (ACL) injuries account for almost half of knee ligament injuries^2^. When both occur simultaneously, it presents a very difficult situation for orthopaedists.

Avulsed fractures of ACL should be reattached in one-stage^3^. As for TPF associated with ACL ruptures, it is commonly accepted that ACL should undergo two-stage reconstruction^3–6^. Why not reconstruct ACL in one-stage procedure? In 1978, Schatzker et al.^3^ concluded in their article that “the collateral ligaments are more important than the anterior cruciate, and for the sake of early mobilization we would be prepared to sacrifice an anterior cruciate and carry out a late reconstruction if necessary”. However, this concept has been challenged by the in-depth studies. Early reconstruction of ACL can provide a nurturing environment for the healing of medial collateral ligament (MCL)^7^. An MCL-deficient knee may still be stabilized by remaining structures, particular ACL^8^. For most combined ACL/MCL injuries, reconstruction of the ACL alone can achieve good clinical results^7–9^.

Other views against one-stage reconstruction included that early reconstruction would cause further soft tissue damage to an already injured knee. But with the advancement of technology for soft tissue protection, these problems have gradually been overcome^10,11^. Bennett and Browner^4^ believed that obtaining bone-patellar tendon-bone graft would interfere with the fracture pattern. Currently, both hamstring and peroneus longus autografts^12,13^ and allografts^14^ have achieved good clinical outcomes.

Meanwhile, two-stage reconstruction has limitations. Some studies had indicated that the most unsatisfactory outcomes of TPF were due to the anterior instability^3,5,10,11^. Two-stage reconstruction has a high incidence of meniscus injury, which leads to secondary changes of the bone and joint^15^ and at least 1-year loss in activity, which also leads to a reduction bone mineral density in the knee^16^.

However, balancing the relationship of tibial tunnel (TT), a conduit that contains and fixes the ACL graft, with fracture and implants is challenging. Many accepted opinions believe that implants will restrict TT and the strength of TT will be insufficient due to the fracture. Nonetheless, there is a lack of available literature specifically demonstrating the infeasibility of one-stage reconstruction.

Finite element analysis (FEA) has been used in orthopaedics for more than two decades and considered as a optimization technique to guide clinical decision-making and to effectively predict the displacement and stress of object under load^17^. In the present study, FEA was used to explore whether the presence of fracture and implants will weaken and restrict the TT.

## Materials and methods

This study was approved by the institutional review board of Chinese People’s Liberation Army General Hospital and in accordance with the ethical standards of Declaration of Helsinki. Written informed consent was provided for the study. A female (age: 23 years 7 months old, height: 171 cm, weight: 57 kg) was enrolled with no previous history of fracture or osteoarthritis, and an average weekly activity level of > 2 h. A computed tomography (CT) scan with 0.5-mm full-length tibia and fibula was obtained.

The CT data were imported into Mimics (Materialize Company, Leuven, Belgium) in Digital Imaging and Communications in Medicine(DICOM) format and then reconstructed in three-dimensional images. Next, the model was input into the Geomagic Studio 2013 (Geomagic, Research Triangle Park NC, USA) in STL format and smoothing, wrapping and segmenting cortical and cancellous bone were defined. A rough measurement of the cortical bone thickness of about 2–4 mm was obtained from the CT scan, thus, cortical bone thickness was set to be 2 mm artificially.

**Figure 1:**
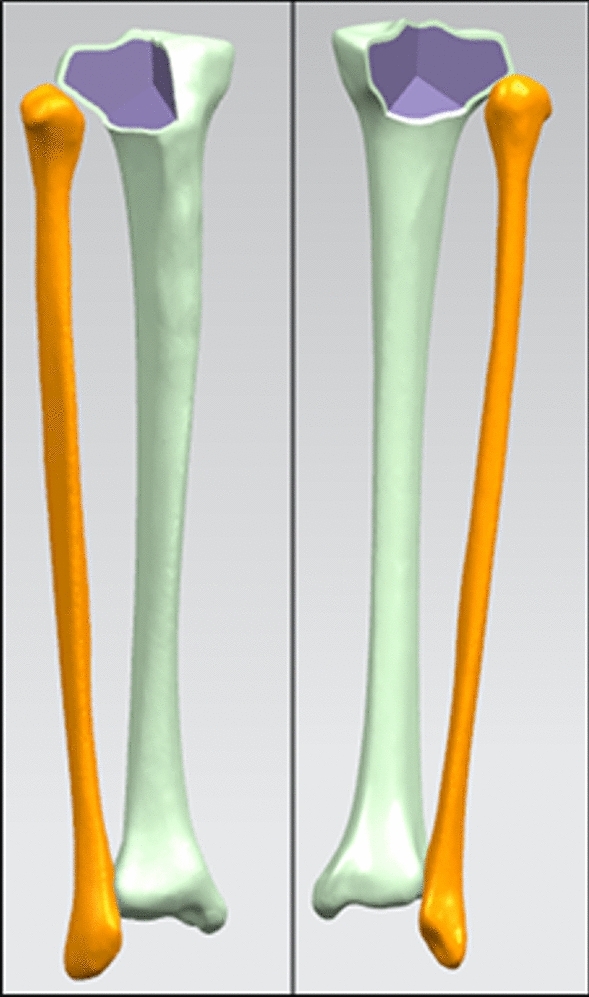
Lateral and posterior column fracture models (created by Solidworks 2018, https://www.solidworks.com/zh-hans).

Next, the above model was imported into Solidworks 2018 (Dassault Systemes Simulia Corp., Providence, RI, USA) in STEP format. The construction of fracture models was based on the “three-column fixation” theory^18^. On the cross section, the tibial plateau was divided in three parts through three lines connecting the three points on the edge of the tibial plateau to the center. One point should be noticed that in article of Luo et al., they use the line from the center of the knee to the most anterior point of the fibular head to separate the lateral and posterior columns^18^. However, through a traditional anterolateral arc incision, the fibular head could be exposed by stripping the upper rear region^19^. Herein, the line from the center of the knee to the posterolateral ridge of the proximal tibia (at approximately the center of the fibular head) was used to separate the lateral and posterior column. As no data available about the thickness of tibial plateau or readymade lateral or posterior column fracture model, so the upper edge of the superior tibiofibular joint (TFJ) was set as the initial plane, three-column connecting lines were made and the bottom of the lateral and posterior fracture surface traversed the tibia at the bottom edge of superior TFJ, then the lateral column fracture (LCF) model, posterior column fracture (PCF) model, and lateral-posterior column fracture (LPCF) model consisting of two fragments were created (Fig. 1).

Next, a cylinder, 1-cm in diameter, was built to simulate TT. The cylinder was positioned at the plane of midpoint between tibial tuberosity and posteromedial ridge and the exit was located slightly behind ACL tibial insertion to prevent interference with the intercondylar roof. The shortest distance between the cylinder and the LCF was about 1 mm and that between the TT and the PCF was about 2 mm. Intact tibial with the TT (ITTT), LCF with the TT (LCFTT), PCF with the TT (PCFTT), and LPCF with the TT (LPCFTT) models were obtained through original models subtracting aforementioned cylinder to stimulate the condition that open reduction and internal fixation in a one-stage ACL reconstruction surgery for the treatment of tibial plateau fracture.

Plates were constructed using bone surfaces and their isometric offset surfaces cutting the solid extrude of plates to simulate complete fits of plates and models. Screws were modeled using cylinders with a diameter of 3.5 mm. As for the LCF and LCFTT, inverted L-shaped plates (80 mm in height, 30 mm in length, 10 mm in width, 4 mm in thickness) were used. Three screws were used in the plateau plane. For the LCF, plateau screws were arranged in parallel, while for LCFTT, the last two screws were adjusted backward by 15°simulating polyaxial screw^20,21^.

T-shaped plates (height: 80 mm, length: 40 mm, width: 10 mm, thickness: 4 mm) were used for PCF and PCFTT. The position of plate was slightly lowered due to the posterior tibial slope angle. Four screws were used in plateau plane. For the PCF, plateau screws were arranged in parallel, while for the PCFTT, the inner two screws were slightly adjusted inward. As for the LPCF and LPCFTT, the two plates and the plateau screws were combined. All plates had one buttress screw and two distal screws.

The models were input into Abaqus 6.14 (Dassault Systemes Simulia Corp., Providence, RI, USA). Implants, including plates and screws, were stimulated Ti6Al4V. Both implants and bone were assumed elastic, linear, and composed of isotropic materials. The elastic modulus of cortical bone, cancellous bone, and implant were 13GPa, 126 MPa, 114GPa respectively, and the respective Poisson’s ratios were 0.30, 0.30, and 0.34^22–24^. The fibula was bonded within the area of proximal tibial of non-fractured bone by multi-point constraints. The distal part of tibia and fibula was fixed without displacement. Friction coefficient between fragments was 0.4^25^ and that between screws and cancellous bone was 0.8. Screws were fixed to cortical bone by sharing the same nodes of elements. The plate was bonded with screws by mimicking locking compression plate and contact surface between plate and bone was assumed to be smooth (see Supplemental Table 1 for all the connection).

**Figure 2:**
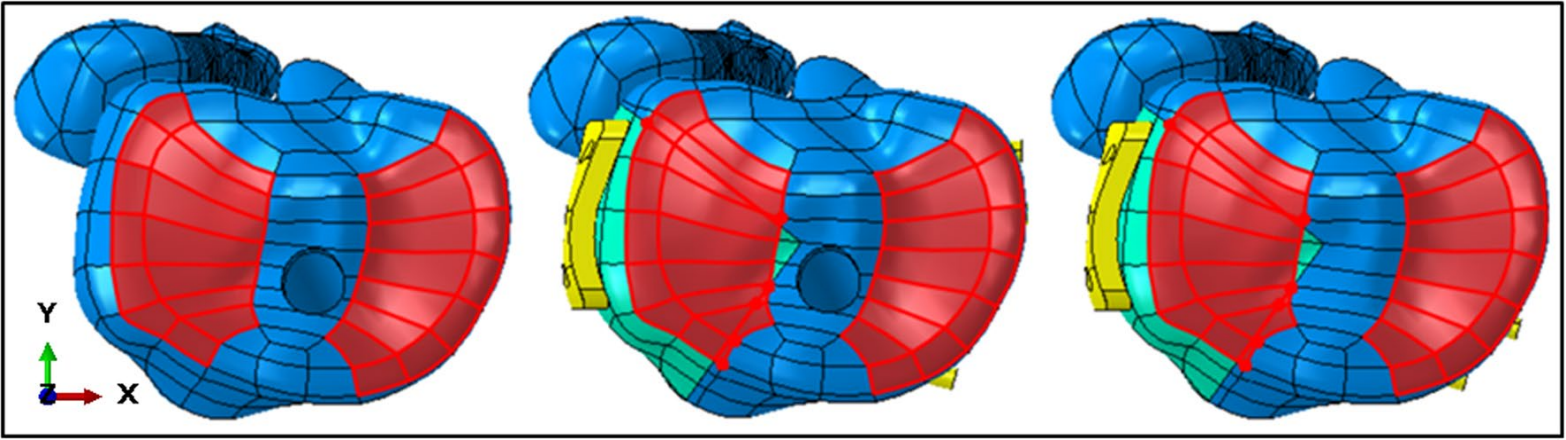
Load area of ITTT, fracture models with tibial tunnel and fracture models without tibial tunnel (take lateral column fracture as an example, created by Abaqus 6.14, https://www.3ds.com/zh/products-services/simulia/products/abaqus/).

The load area was set as the size of a concave formed by connecting line between anterior and posterior horn of medial and lateral meniscus (Fig. 2). 60% force was applied on the medial compartment, and 40% force on the lateral. 80 kg bipedal static standing position was simulated, indicating that a single plateau could withstand about 340 N (80 kg × 9.8 N/kg × 85.6% × 0.5)^23,26^.

For ease of convergence and calculation, tetrahedral four-node elements were used to mesh bones and plates; tetrahedral eight-node elements with reduced integration were used to mesh screws. Element size was three. There were refinements between screws and holes and there was no sharp discontinuity leading to unrealistically high stress concentration. A mesh verification analysis was performed and showed the satisfactory results.

All jobs were submitted and the corresponding plot contours were output. Bearing the capacity and elastic modulus of cortical and cancellous bone are apparently different, they should not be analyzed as a whole as their Von Mises stresses will vary widely. Since cortical bone strength was significantly higher than that of cancellous bone and in clinical situation, as for failure of TT, most of these cases were due to the collapse of the cancellous bone, for this reason, so only the cancellous bone was analyzed here. If external force causes a fracture through TT, it is a completely different matter and were not included in this study. The plate and the screws were analyzed as a whole. For the accuracy of stress and displacement analysis, Von Mises stress on the nodes and displacement on the elements were extracted and the extracted data were the difference before and after the application of force. Since stress and displacement are vectors, all the x-axis (from right to left), y-axis (from anterior to posterior), z-axis (from bottom to top) and absolute values were analyzed (axis of the coordinate system was showed in Fig. 2).

### Statistical analysis

Von Mises stress and displacement were expressed as the mean ± standard deviation. The Student’s t test was used to compare Von Mises stresses and displacements of cancellous bone and implants between corresponding models and of TT between ITTT and matching fracture models with TT. All tests were 2-tailed and p-value < 0.05 was regarded as statistically significant. All statistical analyses were performed using GraphPad Prism 8.0.2 (GraphPad Software, San Diego, CA, USA).

### Ethical review committee statement

This study was approved by the institutional review board.

## Results

**Figure 3:**
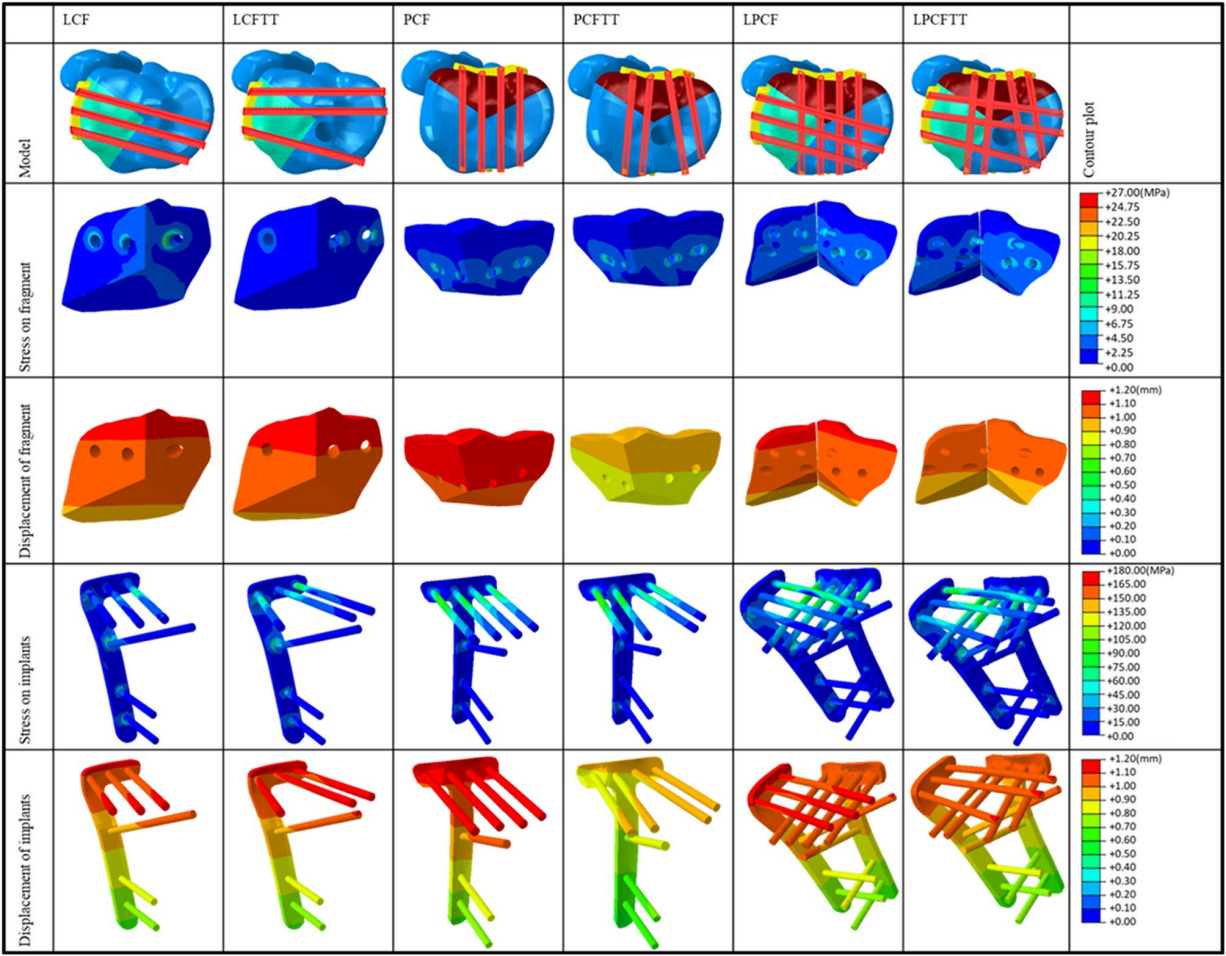
Corresponding plot contours of Von Mises stress and displacement of fragment and implants between fracture models with and without TT and deformation scale factor for each model was uniform and the value of it was ten (created by Abaqus 6.14, https://www.3ds.com/zh/products-services/simulia/products/abaqus/).

Corresponding contourplots and data of Von Mises stresses and displacements of fragment and implants were shown in Fig. 3 and Table 1. The numbers of extracted nodes and elements are shown in parentheses inside table. Almost all differences in parameters used to compare models with TT and matching models without TT were statistically significant. However, differences between matching models were minimal and displacements of fragments of all models were < 2 mm. Besides, fragment displacement of PCFTT and LPCFTT showed even smaller results than corresponding models. Displacement of implants also had similar performance.

**Table 1:** Von Mises stress and displacement of cancellous fragment and implants of fracture models with and without TT.

	Lateral		Posterior		Lateral-Posterior	
	LCF	LCFTT	PCF	PCFTT	LPCF	LPCFTT
Stress on fragment (x-axis)	− 0.06 ± 0.84	− 1.38 ± 0.67	− 0.82 ± 0.58	*− 0.78* ± *0.60*	− 0.61 ± 0.73	− 0.81 ± 0.85
Stress on fragment (y-axis)	− 0.60 ± 0.57	− 0.70 ± 0.62	− 1.57 ± 0.79	*− 1.58* ± *0.86*	− 1.12 ± 0.83	*− 1.06* ± *0.93*
Stress on fragment (z-axis)	− 0.68 ± 0.59	*− 0.38* ± *0.87*	0.22 ± 1.17	0.28 ± 1.24	0.19 ± 1.25	0.28 ± 1.40
Stress on fragment (absolute)	1.52 ± 1.21 (39,390)	1.91 ± 0.02 (37,102)	2.68 ± 1.07 (27,889)	2.79 ± 1.12 (25,495)	2.59 ± 1.12 (78,310)	2.76 ± 1.19 (68,609)
Displacement of fragment (x-axis)	0.56 ± 0.01	*0.55* ± *0.02*	0.59 ± 0.02	*0.48* ± *0.02*	0.56 ± 0.02	*0.53* ± *0.02*
Displacement of fragment (y-axis)	− 0.92 ± 0.02	− 0.94 ± 0.02	− 0.94 ± 0.01	*− 0.75* ± *0.01*	− 0.90 ± 0.03	*− 0.88* ± *0.02*
Displacement of fragment (z-axis)	0.00 ± 0.04	0.01 ± 0.04	− 0.00 ± 0.04	− 0.01 ± 0.03	0.00 ± 0.05	*0.00* ± *0.05*
Displacement of fragment (absolute)	1.08 ± 0.02 (7696)	1.09 ± 0.02 (7273)	1.12 ± 0.02 (5649)	*0.90* ± *0.02* (5195)	1.06 ± 0.03 (16,079)	*1.03* ± *0.02* (14,813)
Stress on implants (x-axis)	5.68 ± 11.91	8.92 ± 15.33	− 0.79 ± 4.99	− 0.48 ± 4.69	4.83 ± 13.64	*4.33* ± *12.98*
Stress on implants (y-axis)	− 0.18 ± 6.07	*0.03* ± *5.70*	10.57 ± 19.30	15.66 ± 22.79	5.41 ± 15.19	8.82 ± 18.79
Stress on implants (z-axis)	0.62 ± 10.24	*0.54* ± *9.79*	− 1.42 ± 8.27	− 1.76 ± 7.41	0.15 ± 9.62	*− 0.03* ± *9.04*
Stress on implants (absolute)	12.38 ± 11.17 (10,524)	15.31 ± 14.04 (11,344)	17.26 ± 17.32 (12,776)	22.17 ± 20.74 (17,091)	17.58 ± 16.95 (24,975)	19.87 ± 18.59 (28,921)
Displacement of implants (x-axis)	0.51 ± 0.08	0.51 ± 0.08	0.52 ± 0.09	*0.44* ± *0.06*	0.51 ± 0.08	*0.49* ± *0.07*
Displacement of implants (y-axis)	− 0.86 ± 0.10	− 0.88 ± 0.10	− 0.88 ± 0.11	*− 0.73* ± *0.07*	− 0.84 ± 0.10	*− 0.83* ± *0.09*
Displacement of implants (z-axis)	− 0.04 ± 0.08	− 0.04 ± 0.08	− 0.06 ± 0.08	*− 0.05* ± *0.06*	− 0.05 ± 0.08	*− 0.05* ± *0.08*
Displacement of implants (absolute)	1.00 ± 0.13 (6856)	1.02 ± 0.13 (8626)	1.03 ± 0.14 (9057)	*0.85* ± *0.09* (13,674)	0.99 ± 0.13 (18,440)	*0.97* ± *0.11* (22,907)

The content inside parentheses was the number of extracted nodes or elements. Data were expressed as mean ± standard deviation. The values of fracture models with TT were compared with corresponding models without TT. The unit of stress and displacement is MPa and mm respectively. The data of corresponding models were not statistically significant or the models with TT were statistically significant (P < 0.05) from the matching models without TT were shown in italics.

In terms of stresses on fragments, all models without TT showed lower stresses than those of corresponding models with TT, but their differences were also small. As for stress concentration, no fracture models with TT showed significant stress concentration, however LCF showed a slightly stress concentration located at the junction between last plateau screw and fracture line with a maximum of 27.5 MPa. The stress applied on implants showed the same phenomenon.

**Figure 4:**
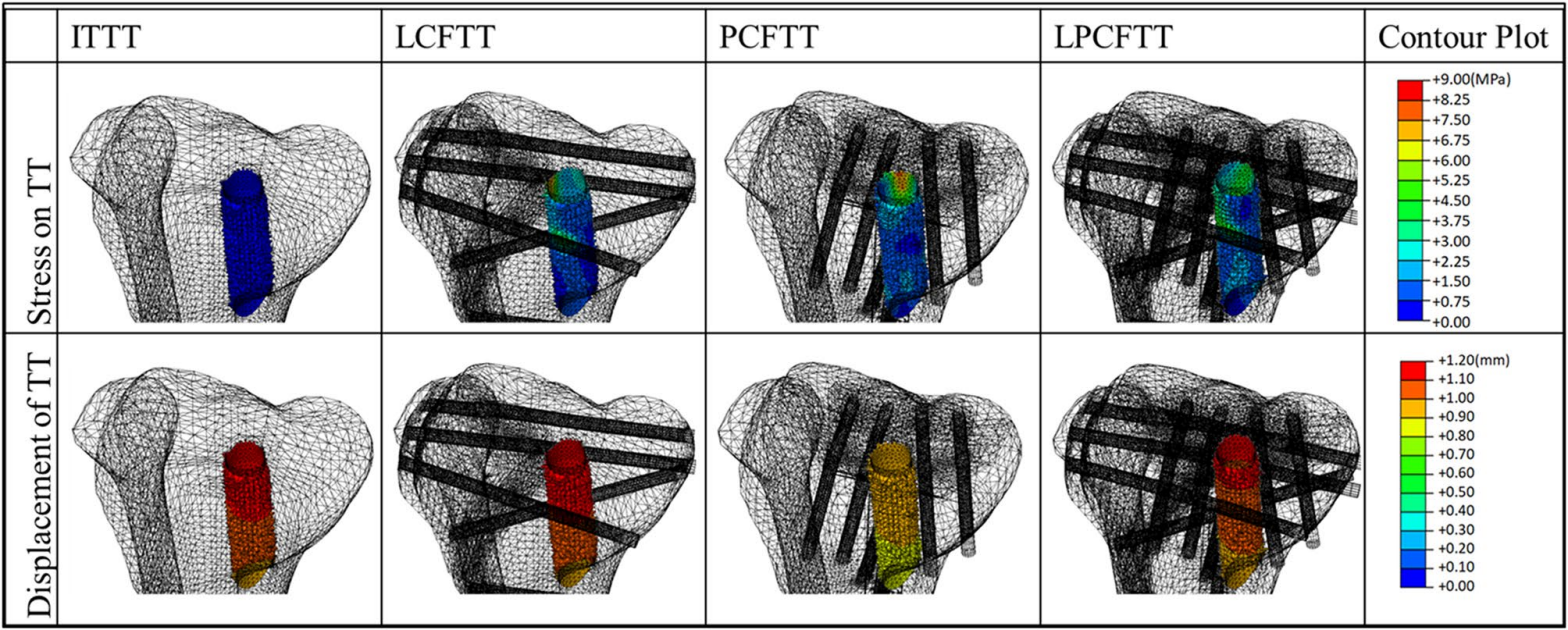
Corresponding plot contours of Von Mises stress and displacement of TT and deformation scale factor for each model was uniform and the value of it was one (created by Abaqus 6.14, https://www.3ds.com/zh/products-services/simulia/products/abaqus/).

Corresponding contourplots and data of Von Mises stresses and displacements of TT were shown in Fig. 4 and Table 2. For stress parameters, the values of TT in fracture models were almost one order of magnitude higher than those in ITTT. The maximum Von Mises stress of TT in ITTT was 0.45 MPa, located in trailing edge of entrance of TT. The maximum of LCFTT was 8.62 MPa, located in outer edge of exit of TT. The maximum of PCFTT was 8.54 MPa, located in trailing edge of exit of TT. Maximum of LPCFTT was 5.50 MPa, located in posterosuperior part of TT. However, displacements showed completely different phenomenon than stresses. There were no statistically significant differences in displacement of TT between ITTT and LCFTT (p = 0.062). Displacement of TT in PCFTT and LPCFTT showed smaller results than that of ITTT.

**Table 2:** Von Mises stress and displacement of TT between ITTT and fracture models with TT.

	ITTT	LCFTT	PCFTT	LPCFTT
Stress (x-axis)	0.01 ± 0.06	− 0.16 ± 0.83	0.69 ± 1.98	− 0.72 ± 0.88
Stress (y-axis)	− 0.00 ± 0.03	0.83 ± 1.61	− 0.13 ± 0.67	− 0.46 ± 0.78
Stress (z-axis)	− 0.17 ± 0.09	0.30 ± 0.78	0.10 ± 0.69	0.51 ± 0.87
Stress (absolute)	0.21 ± 0.07 (1402)	1.69 ± 1.33 (1402)	1.82 ± 1.33 (1305)	2.05 ± 1.13 (1374)
Displacement (x-axis)	0.56 ± 0.04	*0.55* ± *0.03*	*0.47* ± *0.03*	*0.53* ± *0.04*
Displacement (y-axis)	− 0.92 ± 0.05	− *0.93* ± *0.05*	− *0.78* ± *0.04*	− *0.89* ± *0.05*
Displacement (z-axis)	− 0.13 ± 0.02	− *0.13* ± *0.02*	− *0.11* ± *0.02*	− *0.12* ± *0.03*
Displacement (absolute)	1.08 ± 0.06 (735)	*1.09* ± *0.06* (735)	*0.92* ± *0.05* (686)	*1.04* ± *0.06* (721)

The content inside parentheses was the number of extracted nodes or elements. Data were expressed as mean ± standard deviation. The values of the fracture models with TT were compared with those of ITTT. P < 0.05 and the better results of each fracture models are indicated as italics.

**Figure 5:**
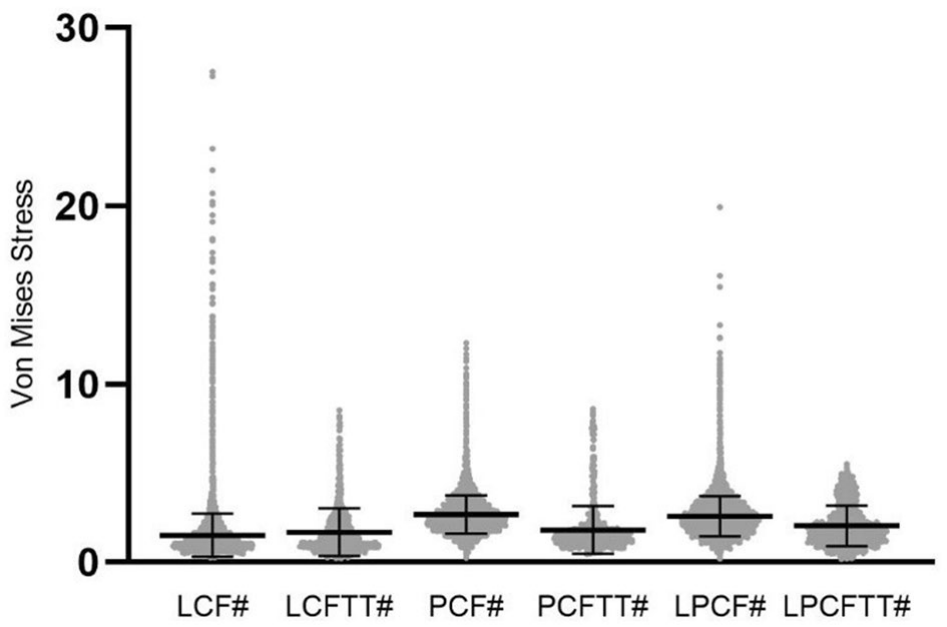
Data dispersion of absolute values of Von Mises stress between TT in fracture model with TT and fragment corresponding fracture model without TT. The unit of Von Mises stress is MPa. LCF# means the stress of fragment of LCF, LCFTT# means the stress of TT in LCFTT, PCF# means the stress of fragment of PCF, PCFTT# means the stress of TT in PCFTT, LPCF# means the stress of fragment of LPCF, LPCFTT# means the stress of TT in LPCFTT.

The stress of TT in fracture models were also compared with the stress of fragments in corresponding fracture models without TT and data dispersion was shown in Fig. 5. Except the mean stress values of TT in LCFTT was higher than that of fragment in LCF (about 0.17 MPa), the mean stress values of TT in other two type were all lower than those of fragments in corresponding fracture models without TT.

## Discussion

To our knowledge, this is the first article in English literature that evaluate the relationship of TT with fracture pattern and implants and we found that although nearly all data were statistically significant, the differences were small and all data were lower than their clinical threshold, which could support ORIF with one-stage reconstruction for treatment of TPF associated with ACL ruptures.

It has frequently been sustained at conferences that TPF associated with ACL rupture is uncommon. What is the incidence of TPF associated with ACL rupture? The Segond fracture strongly suggested ACL rupture with a positive predictive value between 71 and 100%^27,28^. Peltola et al.^28^ reported 23 TPF with Segond fractures, of which 17.4% were associated with complete ACL rupture. In a report describing 39 TPF, eight were associated with ACL injuries^5^, although the type of injury was not described, the authors concluded that the prognosis of TPF associated with cruciate ligament injury was often poor. Thirty cases of TPF were described in which three cases presented ACL injuries, of which one was a mid-substance injury^4^. A further 20 reported cases of nondisplaced and minimally displaced TPF indicated that two were ACL ruptures^29^. Of 103 cases of TPF subjected to surgery, half of the patients showed an ACL partial tear and 11% had complete tear on MRI analysis^30^. There may not be a large number of TPF associated with ACL rupture, but it is not uncommon and treatment has a decisive influence on patient prognosis.

In a report of 64 lateral tibial plateau occult fracture, half had ACL ruptures^31^. Using MRI on 100 patients with complete ACL tears, Kaplan et al. reported that approximately all had posterolateral occult TPF^32^. A further description of 10 patients with posteromedial TPF showed all were accompanied by ACL tears^33^ and in 25 reported cases of posterolateral TPF, 80% of which with ACL tears^34^. Common mechanisms of ACL rupture include excessive anterior displacement of tibia relative to femur and internal rotation and valgus of knee^35,36^. When force is continuous or too large, theoretically, lateral, posterior or posterolateral TPF will occur, which is also the basis for model-building.

In this study, differences in displacement and stress values of cancellous bone of fragment and implants between models with and without TT were nearly all statistically significant, however, the differences were small and each displacement and stress value was below the clinical threshold, which indicated that displacement of fragment was < 2 mm^17^ and stress of implants was < 2800 MPa^22^. In our case, statistically significant did not necessarily mean clinically significant, thus the above results should not be a reason to avoid one-stage reconstruction.

Displacement and stress of TT in ITTT were compared with those in LCFTT, PCFTT, and LPCFTT. The resulting stress of corresponding models was statistically significant with differences of about one order of magnitude. However, the displacement of TT in fracture models showed the same or smaller results than ITTT. Generally, the relationship between displacement and stress should be linear. We think this situation is due to the load weight on TT combined with the simultaneous load generated by support screws and fixed fragment, based on forces of action and reaction, which was responsible for maintaining the shape of TT and the load generated by support screws was larger than that between cancellous bone and that explains why displacements of PCFTT and LPCFTT were smaller than that of ITTT.

Although limited information is available about the strength of TT, the ultimate strength of proximal tibia has been reported as 5.3 ± 2.9 MPa^37^ and the shear strength of lateral tibial plateau cancellous bone may vary from 2.4 to 5.8 MPa^38^. Using electrical measurements, the yield stress of medial tibial plateau was predicted to be 9.0 ± 3.7 MPa^39^. Although the values given above intersects with data reported in our article, our data showed that stress on TT in fracture models was generally smaller than that on fragment in corresponding fracture models and this is not necessary for full-weight bearing for ORIF or reconstruction in early stages. Following a complete and personalized rehabilitation program, the statistical significance of stress applied on TT should also not be considered a reason to prevent one-stage reconstruction of ACL. Double-tunnel ACL reconstruction has improved its effectiveness^40^. Considering only 1–2 mm between the two tunnels, why could not one-stage reconstruction be performed.

Feasibility of ORIF with one-stage reconstruction for LCF, PCF, and LPCF associated with ACL rupture has been discussed in this study. Isolated medial TPF associated with ACL rupture is very rare. When it occurs, because the plate requires placement either medially or antero-medially, and thus is not recommended for one-stage reconstruction. As for medial column fractures, which involve a break in medial column wal^18^, these can usually be classified as Schatzker IV, V, VI, and in some circumstances can undergo one-stage reconstruction. In a case of Schatzker V and knee dislocation that also presented ACL, posterior cruciate ligament (PCL) rupture as well as posterolateral corner injury were treatment by ORIF with one-stage reconstruction^41^. Surgical details, however, were not described. We previously reported a Schatzker VI fracture with a Segond fracture, presenting a fracture line through the medial column. MRI demonstrated a complete proximal ACL rupture and a chronic avulsion of PCL^42^. Because the soft tissue envelope of medial tibial plateau is thin and an anterolateral plate was already present, in that case, posterior plate showed superior biomechanical advantages^43^, thus a posteromedial plate was applied, which successfully fixed the medial column fracture.

Bearing capacity of cortical and cancellous bone is apparently different, they should not be analyzed as a whole. Since cortical bone strength was Some orthopaedists may be discouraged by the placement of forefront screw of LCFTT. Indeed, the distance between TT and anterior edge of tibial is about 1.5 cm^2,40^, which is sufficient to place a screw. If conditions permit, an experienced and skilled orthopaedist and computer navigation could make the process simple.

As for TPF associated with ACL rupture, we can imagine that the injured limb is very swollen and requires calcaneus traction or external fixation to reduce edema for 1–2 weeks. Reconstruction of < 1 week increased the likelihood of arthrofibrosis compared with reconstruction of > 3 weeks^44^, however, a further study^45^ reported that the injury to surgery time was not related to the timing of surgery. In addition to the latter, the occurrence of arthrofibrosis of knee is also associated with soft tissue damage surrounding the knee, graft choice, surgical technique, postoperative rehabilitation, and genetics. Given the numerous uncertainties, long standing disputes will remain for a consensus on arthrofibrosis^46^.

ACL was not simulated in this study because its stiffness was low and did not strongly impact the results. For TT fixation, cross-pin fixation is a safe method but the position of screws needs to be considered, while interference screw fixation does not require consideration of the position of screws but is somewhat risky. Other methods are available, but deserve further study.

This article has similar limitations to those of similar nature. In our study, bone was presume to be an isotropic, linear, elastic material and screws were replaced by cylinders, which is a time-saving and feasible method^47,48^. Although it has been reported that the elastic modulus can be calculated by average CT value^49^, results reported by different studies are inconsistent^50,51^, in addition, the Poisson’s ratio and the association between bone and screws in recent studies still refer to previous reports, which were not specific. Then we assigned a friction coefficient of 0.8 to the surface between screws and cancellous bone which was first proposed in a study of the friction coefficient between bead-surfaced metals and tibial cancellous bone^52^. However, the density and the strength of cortical and cancellous bone differ and friction coefficients should not be simply approximated. Here a term which is commonly used in mechanics and architecture was involved-pretightening force. In our bone-implant system, when screws are fixed to the holes of plate and cortical bone, it can be considered equivalent to the fixation of nuts and bolts. Under no pressure, the interface will produce pretightening force, which is related to material properties, thread and tightening torque. Tightening torque between the hole of cortical bone and screw could be envisioned as the screw being fastened into the cortical bone after tapping and the bone would have a tendency to rebound after being tightened. When the interface is subjected to pressure, the pretightening force is mainly generated by mutual compression between the surrounding bone and the screws. This is the basic theory that the dynamic compression plate can stabilize the fracture. So, we combined the above-mentioned concepts and defined the contact between cortical bone and screws as fixed together.

Then, FEA is a simulation study and if a validation experiment can be performed, it will likely increase the credibility of results.

Next, artificially segmented fracture models and perfect fit between plate and bone do not represent the actual clinical situation. And distribution of screws may differ from case to case.

Finally, only one CT data was enrolled and only bipedal static standing position was simulated. Although increasing the number of CT data may somewhat reduce the effect of geometry differences on results, yet it is not necessary for full weight bearing or walking immediately after surgery and partial weighting or non-weighting will make the results in this article more reliable without the need for additional CT data, in addition the numbers of extracted nodes and elements are more than several thousands, slight differences would make statistically significant, but it did not represent clinically significant. Above all, the purpose of this study was to provide a new clinical insight for treating TPF associated with ACL rupture rather treatment of a specific case.

## Conclusions

This study explored the relationship between TT and fracture patterns and implants. As for stress and displacement of cancellous bone of fragments and implants of models with and without TT, although most differences in evaluated parameters were statistically significant, differences were small and below the clinical threshold. As for TT, although the differences in stress between three corresponding models were approximately one order of magnitude, the differences in displacement of three corresponding model were similar. Following a complete and personalized rehabilitation program, ORIF with one-stage reconstruction for the treatment of TPF associated with ACL rupture can be considered a feasible method. As for orthopaedists, the principles of AO and BO for fracture treatment and the concepts and techniques of sports medicine are equally important. Only by allowing ourselves to keep improving can we provide better benefit to patients.

## Supplementary Information

Supplementary Table 1.
