# Supplementary material for: A finite element analysis of relationship between fracture, implant and tibial tunnel
Source: Sci Rep. 2021 Jan 19;11:1781. doi: 10.1038/s41598-021-81401-6 (PMC7815705; doi:10.1038/s41598-021-81401-6)
Supplement: Supplementary file 1 — Supplementary Table 1. [file 41598_2021_81401_MOESM1_ESM.docx]

**Supplemental Table 1** All contact properties.

| Item1 | Item2 | Interaction type | Note |
| --- | --- | --- | --- |
| Cortical bone | Cortical bone | Norminal contact property: Exponential pressure-overclosure relationship;  Tangential contact property: friction coefficient:0.4 | Between fractured bone and none-fractured bone |
| Cortical bone | Trabecular | Tie |  |
| Cortical bone | Screws | Tie |  |
| Cortical bone | Metal plate | Contact,no friction |  |
| Trabecular | Trabecular | Norminal contact property: Exponential pressure-overclosure relationship;  Tangential contact property: friction coefficient:0.4 | Between fractured bone and none-fractured bone |
| Trabecular | Screws | Norminal contact property: Exponential pressure-overclosure relationship,  Tangential contact property: friction coefficient:0.8 |  |
| femur | tibia | multi-point constraints(MPC) constraints: Beam |  |
| Metal plate | Screw | Tie |  |
